# High-flow nasal oxygen in infants and children for early respiratory management of pneumonia-induced acute hypoxemic respiratory failure: the CENTURI randomized clinical trial

**DOI:** 10.1007/s44253-024-00031-8

**Published:** 2024-04-01

**Authors:** Sasidaran Kandasamy, Ramachandran Rameshkumar, Thangavelu Sangaralingam, Nedunchelian Krishnamoorthy, N. C. Gowri Shankar, Vimalraj Vijayakumar, Balaji Sridharan

**Affiliations:** 1 Advanced Pediatric Critical Care Centre, Pediatric Acute Care Education & Research (PACER) Unit, Department of Pediatrics, Mehta Multi Speciality Hospitals, Chennai, Tamil Nadu 600 031 India; 2 Division of Pediatric Critical Care, Department of Pediatrics, Jawaharlal Institute of Postgraduate Medical Education and Research (JIPMER), Puducherry, 605 006 India; 3 Present Address: Pediatric Critical Care, Mediclinic City Hospital, Mohammed Bin Rashid University of Medicine and Health Sciences (MBRU), Dubai, United Arab Emirates; 4 Department of Pediatrics, Mehta Multi Speciality Hospitals, Chennai, Tamil Nadu 600 031 India; 5 Department of Research & Academics, Mehta Multi Speciality Hospitals, Chennai, Tamil Nadu 600 031 India; 6 Advanced Pediatric Critical Care Centre, Department of Pediatrics, Mehta Multi Speciality Hospitals, Chennai, Tamil Nadu 600 031 India; 7 Pediatric Acute Care Education and Research (PACER) Unit, Department of Pediatrics, Mehta Multi-Speciality Hospitals, Chennai, Tamil Nadu 600 031 India

**Keywords:** Children, Pneumonia, Respiratory support, Mechanical ventilation

## Abstract

**Objective:**

To compare the effectiveness of early high-flow nasal cannula (HFNC) and low-flow oxygen support (LFOS) in children under 5 years with acute hypoxemic respiratory failure (AHRF) due to severe community-acquired pneumonia in low-middle-income countries.

**Methods:**

An open-label randomized clinical trial enrolled children aged 2–59 months with AHRF due to severe community-acquired pneumonia and randomized into HFNC and LFOS. In the LFOS group, the patient received cold wall oxygen humidified by bubbling through sterile water administered through simple nasal prongs at a fixed flow rate of 2 L/min. In the HFNC group, the patient received humidified, heated (37 °C), high-flow oxygen at a flow rate assigned based on weight range, with a titratable oxygen fraction. The primary outcome was treatment failure in 72 h (escalating the respiratory support method using any modality other than primary intervention).

**Results:**

Data was analyzed intention-to-treat (HFNC = 124; LFOS = 120). Median (IQR) age was 12 (6–20) and 11 (6–27) months, respectively. Treatment failure occurred in a significantly lower proportion in the HFNC group (7.3%, *n* = 9/124) as compared to the LFOS group (20%, *n* = 24/120) (relative risk = 0.36, 95% CI 0.18 to 0.75; *p* = 0.004; adjusted hazard ratio 0.34, 95% CI 0.16 to 0.73; *p* = 0.006). The intubation rate was significantly lower in the HFNC group (7.3%, *n* = 9/124 vs. 16.7%, *n* = 20/120; relative risk = 0.44, 95% CI 0.21 to 0.92, *p* = 0.023). There were no significant differences noted in other secondary outcomes. No mortality occurred.

**Conclusion:**

High-flow nasal cannula oxygen therapy used as early respiratory support in children under 5 years with acute hypoxemic respiratory failure due to severe community-acquired pneumonia was associated with significantly lower treatment failure compared with standard low-flow oxygen support.

**Trial registration:**

CTRI/2016/04/006788. Registered 01 April 2016, https://ctri.nic.in/Clinicaltrials/advsearch.php.

**Supplementary Information:**

The online version contains supplementary material available at 10.1007/s44253-024-00031-8.

## Introduction

Globally, the pneumonia burden among children under 5 years is estimated to be 138 million in 2015, with India carrying 32% of the total load [1, 2] and contributing to 16% of under-5 years mortality [3, 4]. Infections contribute to most community-acquired pneumonia in low-middle-income countries (LMIC) [5]. Though most children are managed on an outpatient basis, hospitalization and oxygen therapy are imperative for those with severe community-acquired pneumonia [6, 7]. The earliest and most accessible method of oxygen administration is low-flow oxygen support (LFOS) through nasal cannula (prong). The heated, humidified, high-flow nasal cannula oxygen (HFNC) therapy is increasingly used in different respiratory pathologies. HFNC therapy is one of the better-tolerated treatment modalities in children and adults with respiratory pathologies [8–10].

Our study aimed to determine the effectiveness of HFNC versus LFOS as early respiratory support in children aged 2 to 59 months with severe community-acquired pneumonia. We hypothesized that HFOS support would be associated with a lower proportion of treatment failure than LFOS support in this group of children.

## Material and methods

An open-label randomized controlled trial was conducted in the pediatric emergency room (ER) and pediatric intensive care unit (PICU) of a tertiary care teaching hospital from May 2016 to February 2019 under the International Clinical Epidemiology Network (INCLEN) childhood pneumonia project. The ethics committees of the study site and the INCLEN ethics committee approved the study protocol. The study was performed following the principles of the Declaration of Helsinki. Children aged 2–59 months who attended the ER with fever, respiratory distress, and tachypnoea [(2–12 months: respiratory rate (RR) ≥ 50/min; 13–59 months: RR. ≥ 40/min)] who were categorizable as severe based on oxygen saturation (SpO_2_) of < 92% in room air or SpO_2_ 92% to 94% with chest indrawing (subcostal/intercostal/suprasternal retraction) and/or depressed sensorium were screened [11]. They underwent chest x-ray and standard respiratory stabilization (face mask oxygen, nebulization with salbutamol plus ipratropium bromide—three times in 13–59 months, two doses of adrenaline in ≤ 12 months as per treating team discretion) for 1 h. Those who persisted in requiring oxygen support were enrolled after obtaining written informed consent from one of the parents or a legally acceptable alternative.

Children with either (*i*) chronic lung disease, (*ii*) bronchial asthma, (*iii*) neuro-muscular disease, (i*v*) hospitalized in the previous 30 days, (*vi*) receiving home ventilation or oxygen support, (*vii*) congenital cardiac or other multiple malformations, or (*vii*) diagnosed to have empyema were excluded.

A web-based, computer-generated, stratified (by age strata, 2–12 months and 13–59 months) block randomization with variable block size was used. The allocation was concealed and kept in sequentially numbered opaque sealed envelopes (SNOSE). The study intervention was not blinded because of the nature of the intervention. However, the person handling the data and the statistician were blinded for intervention assignment until the final analysis.

All other medical interventions (antibiotics, intravenous fluid 5% dextrose in 0.9% saline at 70% of calculated fluid requirement), supportive interventions (nebulization, nasogastric or nasoduodenal feeds, positioning, nursing care), and investigations were kept similar in both groups. Organ function assessments and repeat blood workups were done at the attending physician’s discretion.

*In the LFOS group*, cold wall oxygen was humidified by bubbling through sterile water and administered through simple nasal prongs at a fixed flow rate of 2 L/min (LPM). *In the HFNC group*, humidified, heated (37 °C), high-flow oxygen was started at the flow rate as per child’s weight (2 L/kg/min if weight ≤ 10 kg; 20L + 0.5 L/kg for each kg above 10 kg) using AIRVO_2_™ high-flow oxygen therapy system. Age-appropriate Optiflow™ nasal cannula and other disposables were used to deliver HFNC. Support was started at 60% FiO_2_ and then down-titrated based on the SpO_2_ target of 94% to 98% after 4 h of therapy. All enrolled patients were shifted to the PICU for further management. The unit transport protocol reduced transport time to 10 min or less.

In the first 4 h of treatment, children were maintained nil by mouth and later started on enteral feeds as per standard unit protocol. FiO_2_ escalation above 60% was not allowed, and de-escalation of FiO_2_ up to 30% was allowed in the HFNC group after 4 h based on target SpO_2_, but flow reduction was not permitted until it was decided to wean. Weaning from respiratory support was initiated when the treating physician certified decreased work of breathing, average age-specific respiratory rate with hemodynamic stability for 6 h. In the HFNC group, FiO_2_ decreased to 30% before flow reduction. Furthermore, the flow rate was reduced to five LPM and kept for 4 h to ascertain stability and then switched to low flow at the rate of one LPM for 2 h and stopped. Escalation of respiratory supports (i.e., failure of primary intervention) was based on overall clinical conditions (persistent tachycardia, tachypnea, worsening respiratory distress (RD) score, depressed sensorium, and decreasing SpO_2_). To ensure uniform care, the healthcare providers were equipped with a checklist of standard care components and RD score (Supplementary e-Table 1) [12]. A separate research assistant not involved in clinical care and outcome assessment was assigned for data collection.

Based on pre-trial observations, the following two amendments were made in the study protocol: in any child failing with primary intervention, (i) in the HFNC group, continuous positive airway pressure (CPAP) or Bi-level positive airway pressure (BiPAP) was allowed pre-intubation or escalation of respiratory support; (ii) in the LFOS group, HFNC, CPAP, or Bi-PAP was allowed pre-intubation or escalation of respiratory support; (iii) in either group, rescue steroid (intravenous dexamethasone 0.6 mg/kg/day on day 1, 0.3 mg/kg/day on day 2, 0.15 mg/kg/day for days 3 and 4) was allowed. Safety of the study procedure was ensured by monitoring air leaks, sudden hemodynamic or respiratory worsening events, nasal/nasal bridge injury or ulceration, or bleeds, using the checklist and reporting to the external data and safety monitoring committee. Serious adverse events were defined as life-threatening, fatal, or compromising the neurologically intact outcome at discharge.

Venous blood gas was done for all enrolled children, and arterial blood gas was done at the attending physician’s discretion. The vital signs, chest retraction [mild—subcostal; moderate—intercostal; severe—suprasternal], SpO_2_ (by Life Scope monitor with smart cable technology™ by Nihon Kohden, Japan), and RD score were documented every 30 min and reviewed by the primary treating team. Patient monitoring was continued until the child remained on oxygen support. Fluid overload percentage (% F.O.) was calculated daily [% F.O. = (total fluid intake in liters − total fluid output in liters ÷ admission weight in kilograms) × 100] [13, 14]. All discharged patients were followed up for 30 days.

The primary outcome was treatment failure in 72 h (defined as escalating the respiratory support method using any modality other than primary intervention). The secondary outcomes were (*i*) time to achieve clinical and respiratory stability (normal RR for age; 2–12 months: RR ≤ 50/min; 13–59 months: RR ≤ 40/min), not on vasoactive drugs, no perfusion abnormalities, SpO_2_ ≥ 95% in room air or with 40% FiO_2_ in HFNC group for 6 h), (*ii*) % FO at 24 and 48 h, (*iii*) adverse events, and (*vi*) in-hospital mortality.

### Statistical analysis

McKiernan et al. and Schibler et al. found a reduction of intubation from 23 to 9% and 37 to 7% by HFNC, respectively [15, 16]. With the assumption that baseline treatment failure in the LFOS group was 23% and in the HFNC group was 7% with 1:1 allocation, 90% power, and a two-sided alpha of 5% with an attrition rate of 10%, the required sample size was 120 per group.

All subjects’ data were analyzed according to their assigned group (intention-to-treat analysis). The normality of data was checked with the Kolmogorov-Smirnov *Z* test. The chi-square test (Fisher’s exact test if cell frequency was less than five) was used to compare the proportions. Continuous variables were compared by Student’s *t* test if normally distributed or Mann-Whitney *U* test if not normally distributed. Kaplan-Meier and log-rank test followed by the Cox proportional hazard model was used to adjust the prior for age strata, gender, and severity (by RD score). The adjusted hazard ratio, the relative risk, and the number needed to treat (NNT) with a 95% confidence interval (95% CI) was calculated. All the tests were two-tailed, and a *p*-value < 0.05 was considered statistically significant. Data were analyzed using IBM-SPSS, version 20.0 (SPSS Inc. Chicago, Illinois).

## Results

**Fig. 1:**
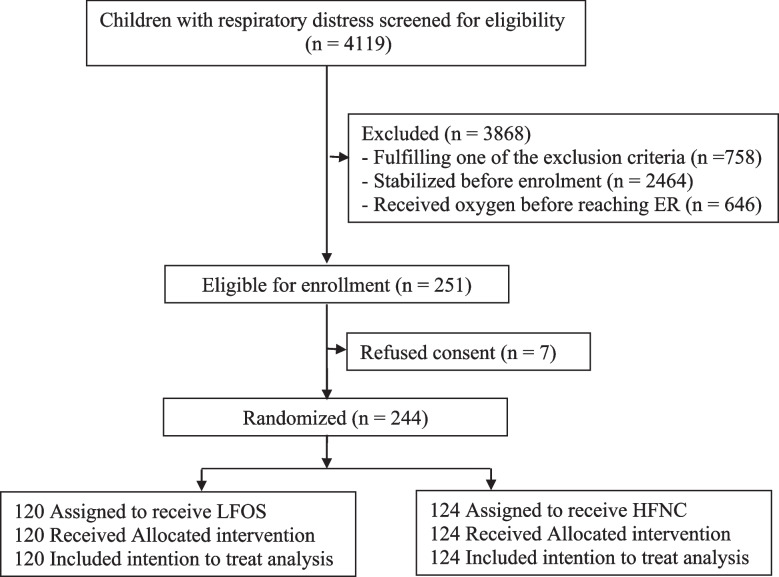
Trial flow. E.R., emergency room; LFOS, standard low-flow oxygen support; HFNC, high-flow nasal cannula oxygen support

The trial flow is depicted in Fig. 1. A total of 244 patients were enrolled (HFNC group, *n* = 124, and LFOS group, *n* = 120). The baseline characteristics are comparable and given in Table 1. All 244 children completed treatment in the same hospital and had a minimum of one telephone follow-up on day 30 of discharge. Four children (*n* = 4/244, 1.6%) had *Streptococcus pneumoniae* in the blood culture, and 58.2% (*n* = 142/244) were positive for one or more tested viruses by real-time polymerase chain reaction (RT-PCR) test. Eighteen-one (33.2%) received sedation during the hospital stay (HFNC: *n* = 50/124, 40.3% vs. LFOS: *n* = 31/120, 25.8%). Sixty-eight (45 vs. 23) received midazolam, seven (2 vs. 5) received triclofos, and six (3 vs. 3) received dexmedetomidine.

**Table 1:** Baseline characteristics of two study groups at the time of enrollment

*Parameter*	*HFNC group (n* = *124)*	*LFOS group (n* = *120)*
Age, months^a^	12 (6–20)	11 (6–27)
Age group, *n* (%)		
2–12 months	62 (50)	66 (55)
3–59 months	62 (50)	54 (45)
Male: female, *n* (%)	84 (68):40 (32)	77 (64):43 (36)
Weight, *z* score^b^	- 0.96 (1.40)	- 1.09 (1.28)
Length, *z* score^b^	-0.13 (2.20)	-0.24 (2.57)
Body mass index^b^	15.5 (5.8)	15.3 (4.6)
Day of illness^a^	3 (2–5)	3 (2–5)
Duration of fever, days^a^	1.5 (1–4)	1 (0–4)
Respiratory distress score	8.7 (1.1)	8.8 (1.2)
Temperature, °F	99.5 (1.1)	99.7 (1.4)
Chest retraction, *n* (%)	124 (100)	120 (100)
Grunting, *n* (%)	39 (31.5)	36 (30)
Stridor, *n* (%)	3 (2.4)	6 (5)
Saturation (%), category, *n* (%)		
Less than 92% with chest retraction^c^	32 (25.8)	22 (18.3)
92–94% with chest retraction^c^	92 (74.2)	98 (81.7)
Time to randomization, hours^b^	2.55 (0.44)	2.47 (0.43)
Heart rate per minute^b^		
2–12 months	156 (22)	157 (18)
3–59 months	154 (21)	149 (20)
Respiratory rate per minute^b^		
2–12 months	66 (13)	61 (11)
3–59 months	60 (15)	55 (12)
pH^b^	7.39 (0.08)	7.41 (0.09)
PCO_2_, mm Hg^b^	36.3 (9.4)	35.0 (8.6)
Bicarbonate, mEq/L^b^	20.6 (6.5)	20.7 (5.9)
Initial intravenous fluid, *n* (%)		
5% dextrose in normal saline	61 (49)	53 (44)
Normal saline	11 (9)	17 (14)
Balanced salt solution (BSS)	52 (42)	50 (42)
Empiric antibiotics, *n* (%)	115 (93)	103 (86)
CRP, positive, *n* (%)	62 (50)	62 (52)
CRP, mg/L among positive patients^a^	19.5 (11.0–32.0)	22.5 (10.0–48.0)
CRP, mg/L, all patients^a^	4.0 (3.0–22.5)	4.0 (2.0–18.0)
Hemoglobulin, gm%^b^	10.8 (1.6)	11.2 (2.9)
Viral respiratory panel workup, *n* (%)		
Respiratory syncytial virus	14 (11.3)	20 (16.7)
Influenza	28 (22.6)	24 (20)
Mixed	18 (14.5)	10 (8.3)
Others	16 (12.9)	12 (10)
No yield	48 (38.7)	54 (45)

*HFNC* high-flow nasal cannula oxygen support, *LFOS* low-flow oxygen support, *F* degrees Fahrenheit, *RD score* respiratory distress score, *PC*O_2_ partial pressure of carbon dioxide (venous), *IQR* interquartile range, *SD* standard deviation, *CRP* C-reactive protein. The percentage of patients, vitals, and blood test parameters were rounded to the nearest number wherever appropriate

All values are in number and/or with the percentage in paracentesis except

^a^median (IQR)

^b^mean (SD)

^c^One or more findings may present in the same patient (subcostal/intercostal/suprasternal retraction). *p*-value: not significant

**Fig. 2:**
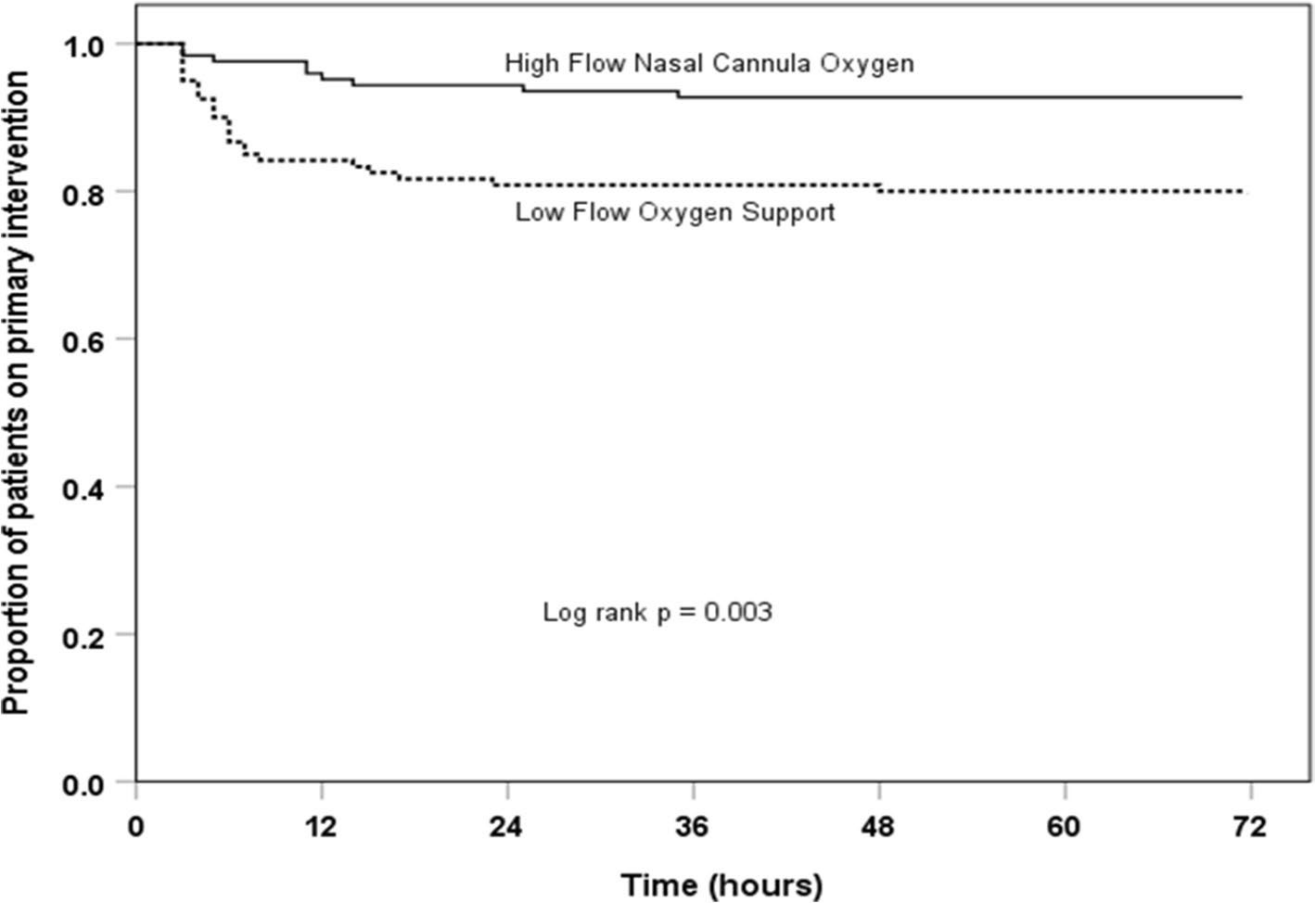
Kaplan-Meier curve showing the treatment failure between two study groups. LFOS, standard low-flow oxygen support; HFNC, high-flow nasal cannula oxygen support

The treatment failure occurred in a significantly lower proportion in the HFNC group (7.3%, *n* = 9/124) as compared to the LFOS group (20%, *n* = 24/120) (relative risk = 0.36, 95% CI 0.18 to 0.75, *p* = 0.004) (Table 2; Fig. 2). The hazard ratio of the treatment failure was significantly lower by 66% in the HFNC group (adjusted hazard ratio 0.34, 95% CI 0.16 to 0.73; *p* = 0.006). The number needed to treat (NNT) was 8 (95% CI 5 to 24). Among treatment failure, the mean (SD) RD score was similar between the two study groups [9.0 (1.3) vs. 8.9 (1.3); *p* = 0.937]; saturation of less than 92% with chest retraction was 4/9 vs. 6/24, and saturation of 92–94% with chest retraction was 5/9 vs. 18/24 (*p* = 0.400). Among treatment failure, all received dexamethasone, and nine patients in the HFNC group (7.3%) and 20 patients in the LFOS group (16.7%) were intubated despite HFNC rescue; four patients in the LFOS group were rescued from intubation by HFNC rescue (relative risk = 0.44, 95% CI 0.21 to 0.92, *p* = 0.023). The clinical criteria met at the decision to intubation were persistent tachycardia in 100% (*n* = 9/9) vs. 90% (*n* = 18/20), persistent tachypnea in 77.8% (*n* = 7/9) vs. 100% (*n* = 20/20), and decreasing SpO_2_ in 100% (*n* = 9/9) vs. 100% (*n* = 20/20) in the HFNC and LFOS groups respectively. The treatment failure occurred significantly early in the LFOS group compared to the HFNC group (Fig. 2). No significant difference was noted in the first 72 h of the mean (S.D.) R.D. score [6.4 (1.4) vs. 6.5 (1.7); *p* = 0.552]. No significant difference was noted in the other secondary outcomes (Table 2). There was no in-hospital mortality, and no trial-related adverse events occurred.

**Table 2:** Outcome variables in the two study groups

*Parameter*	*HFNC group (n* = *124)*	*LFOS group (n* = *120)*	*p-value*
*Primary outcome*			
Treatment failure, *n* (%)	9 (7.3)	24 (20)	0.004^*^ (relative risk 0.36, 95% CI 0.18 to 0.75)
Treatment failure by age strata, *n* (%)			
2–12 months	4/62 (6.5)	12/66 (18.2)	0.045^†^ (relative risk 0.35, 95% CI 0.12 to 1.02)
13–59 months	5/62 (8.1)	12/54 (22.2)	0.032^*^ (relative risk 0.35, 95% CI 0.14 to 0.96)
*Secondary outcomes*			
Clinical stability, hours^a^	109 (84–138.5)	120.5 (61–156.5)	0.818^§^
Respiratory stability, hours^a^	107 (82–135)	108 (58.5–145.5)	0.496^§^
Fluid overload (%FO)^b^			
At 24 h	2.11 (2.24)	1.95 (2.18)	0.573^¶^
At 48 h	1.82 (2.35)	1.53 (2.12)	0.313^¶^
Adverse events, *n* (%)	-	-	-
In-hospital mortality, *n* (%)	-	-	-

*LFOS* low-flow oxygen support, *HFNC* high-flow nasal cannula oxygen support, *IQR* Interquartile range, *SD* standard deviation, *95% CI* 95% confidence interval

All values are in number and/or with the percentage in paracentesis except ^a^median (IQR) and ^b^mean (SD)

^*^Chi-square test

^†^Fisher’s exact test

^§^Mann-Whitney *U* test

^¶^Student *t* test

## Discussion

In our study, early initiation of HFNC as a primary respiratory support modality was found to have significantly lower treatment failure compared to those on standard LFOS. We have not observed any difference among the groups in secondary outcomes. There was a probability of difference in etiology and clinical manifestations among the 2–12 months and 13–59 months age groups [5, 17, 18]. We compared the primary outcome in these pre-specified age strata. We did observe significant differences in treatment failure among the age group strata. However, the event rate in these groups was not adequately powered to study the primary outcome.

Though the varying incidence of air leak events had been documented [19, 20] in previous studies, we did not observe any significant HFNC-attributable adverse events when we strictly followed a protocolized flow prescription method without any flow escalation based on clinical status. Our observation confirmed that providing high-flow support safely in a mixed setting of the ER and PICU is feasible when we strictly follow a protocolized prescription order and ensure proper monitoring by trained personnel.

Treatment failure in our study compared to other recently published trials in bronchiolitis and childhood pneumonia [21, 22], where it had been differentially defined as the duration of oxygen support [23], length of stay [10, 24], the need for non-invasive or invasive respiratory escalation [25, 26], day-28 mortality [26, 27], worsening respiratory distress and discomfort [28], or a composite outcome definition for treatment failure including more than one above mentioned outcome measures [26, 29]. A meta-analysis by Luo et al. of HFNC vs. standard oxygen or nasal-CPAP in children with acute lower respiratory tract infection (bronchiolitis, pneumonia), hypoxemia, and respiratory distress aged 29 days to 5 years found that HFNC significantly reduced the treatment failure in mild hypoxia patients (risk ratio = 0.49, 95% CI 0.40–0.60). However, HFNC was associated with an increased risk of treatment failure in infants 1–6 months with severe hypoxia (risk ratio = 1.77, 95% CI 1.17–2.67) and no difference in intubation and mortality rate [26]. The COAST study involving African children aged 28 days to 12 years with severe pneumonia found no difference in the primary endpoint (mortality at 48 h) between HFNC (1.1%) and LFOS (2.5%). Treatment failure (saturation < 92% with respiratory distress) at 48 h was lower in HFNC, but the difference was not statistically significant (adjusted odds ratio = 0.75, 95% CI 0.40–1.41). However, the study was stopped prematurely by the trial steering committee as a result of deemed permissive hypoxemia unethical [30]. Mild to moderate acute hypoxic respiratory failure (AHRF) due to different respiratory pathology in children aged 1–4 years showed HFNC associated with prolonged hospital stay as compared with standard oxygen therapy. The possible reasons could be slow weaning with no additional observation after the cession of HFNC, the familiar weaning process of standard oxygen therapy, and no data recorded during the weaning process in addition to different pathologies of AHFR [10]. Though these studies are similar to our study setting and intervention (HFNC), the differences are age group (2–59 months), the weaning process, and the primary outcome (treatment failure at 72 h). Primary outcome difference was also noted among different age groups, and the requirement for intubation was significantly lower in the HFNC group. Avoiding mechanical ventilation may be the most meaningful public health-relevant justification for efforts to create facilities at the ER to initiate such therapy in LMIC, where invasive ventilation facilities are limited. We were able to rescue 4/24 by HFNC in LFOS-failed patients. Failure of HFNC as rescue therapy in LFOS-failed patients in our study raises concern about the “time to decide therapy failure” and whether it is too late to be rescued by HFNC. “Rescue HFNC” in LFOS-failing children included a concomitant administration of steroids as well; this cautioned us to conclude favorably or against the efficiency of rescue HFNC based on our observations. Though the intubation rate was significantly lower in HFNC compared to LFOS (7.3% vs. 16.7%), it was in contrast to recent study results. The meta-analysis involving children with respiratory distress due to different respiratory pathologies found no difference in the probability of intubation between HFNC and LFOS (risk ratio = 0.97, 95% CI 0.56–1.68). A similar study setting by Liu et al. reported that treatment failure requiring intubation was similar in the HFNC (14%) and in the CPAP (10%) group in children less than 2 years with mild-moderate respiratory failure due to pneumonia [29].

Our study was one of the few to evaluate HFNC vs. LFOS as early respiratory support in children under 5 years with severe pneumonia conducted in an LMIC. The trial followed pragmatic and more inclusive eligibility criteria about under-5 age group children presenting with acute respiratory distress in the ER, used a standard screening protocol to identify those with severe pneumonia requiring hospitalization, and examined the feasibility of administrating HFNC support in a mixed clinical setting alongside the evaluation of therapy effectiveness. Though our cohort was not the “severely hypoxic children” and our SpO_2_ targets were more stringent than WHO recommendations [26], and the bronchiolitis of infancy discharge study (BIDS) study observation [31], our standardized pre-enrolment stabilization maneuver helped us to select children who were severe enough to require oxygen support and hospitalization at the same time safe enough to start the therapy in ER. Failed patients required intubation, and there was no scope for variability in the escalation of care based on a clinical decision, which was documented to be 34% in another large multi-centric trial by Franklin et al. [25]; at the same time, this could have been attributed to the relatively delayed trigger point to initiate HFNC rescue. The limitation of the study was that it was a single-center study and could not be extrapolated to pediatric wards or very high-load ERs with limited resources. The allocation was not blinded because of the given nature of the intervention. The study was conducted close to a real-world scenario in terms of most of the clinical and methodological perspectives. Multi-centric trials involving community hospitals are needed to substantiate our observations and generalizability.

## Conclusion

The study concludes that high-flow nasal cannula oxygen therapy used as early respiratory support in children under 5 years with acute hypoxemic respiratory failure due to severe community-acquired pneumonia was associated with significantly lower treatment failure compared with standard low-flow oxygen support.

### Supplementary Information

**Additional file 1: e-Table 1.** Respiratory Distress Score [RDS].

## Data Availability

SK is the guarantor of the paper. S.K. has full access to data, which shall be shared upon request.
