# Supplementary material for: High-flow nasal oxygen in infants and children for early respiratory management of pneumonia-induced acute hypoxemic respiratory failure: the CENTURI randomized clinical trial
Source: Intensive Care Med Paediatr Neonatal. 2024 Apr 1;2(1):15. doi: 10.1007/s44253-024-00031-8 (PMC10982089; doi:10.1007/s44253-024-00031-8)
Supplement: Supplementary file 1 — Additional file 1: e-Table 1. Respiratory Distress Score [RDS]. [file 44253_2024_31_MOESM1_ESM.doc]

Supplementary Appendix

Title: High flow nasal oxygen in infants and children for early respiratory management of pneumonia-induced acute hypoxemic respiratory failure: The CENTURI randomized clinical trial

e-Table 1. Respiratory Distress Score [RDS]

| **Clinical parameter** | **Score-1** | **Score-2** | **Score-3** |
| --- | --- | --- | --- |
| **Oxygen saturation** | Mild hypoxemia, SpO2 90–93% during crying only | Mild hypoxemia, SpO2 90–93% at rest | Hypoxemia, SpO2 < 90% |
| **Chest wall retraction** | None or minimal | Moderate chest wall retraction | Marked chest wall retractions, tracheal tug |
| **Respiratory sounds** | None or minimal | Intermittent soft grunting AND/OR nasal flaring | Grunting with every breath and nasal flaring |
| **Feeding** | Normally feeding | Difficulty with feeding  Reduced feeding because of respiratory distress | Unable to feed because of respiratory distress or lethargy |

Interpretation: Mild respiratory distress (score 4-6)

Moderate respiratory distress (score 7-9)

Severe respiratory distress (score 10-12)
